# Control of the medical fitness for work of health care workers on psychiatric leave

**DOI:** 10.1192/j.eurpsy.2023.669

**Published:** 2023-07-19

**Authors:** S. Ernez, D. Brahim, M. Mersni, L. Ben Afia, W. Ayed, N. Mechergui, I. Youssef, N. Ladhari

**Affiliations:** Occupational pathology and fitness for work, Charles Nicolle Hospital, Tunis, Tunisia

## Abstract

**Introduction:**

Absenteeism from work is considered to be a major source of disorganization and professional marginalization. Psychiatric leave is a frequent form of absenteeism in the hospital environment requiring medical control of the ability to work in order to detect certain abusive prescriptions or certain psychological disorders that can be professionally disabling.

**Objectives:**

To draw up the socio-demographic, professional and clinical profile of the health care workers examined within the framework of a medical examination of the aptitude for work following a psychiatric sick leave.

To determine the medical fitness-for-duty decisions in interaction with the prescribed psychiatric leave

**Methods:**

Retrospective descriptive study on the files of health care personnel who had psychiatric leaves and who were examined in a framework of multidisciplinary medical commission of absenteeism carried out in the department of professional pathology and aptitude for work at the Charles Nicolle Hospital of Tunis. The study period was from January 1, 2020, to October 1, 2022

**Results:**

We collected 63 records. The average age was 44.75 years +/-11.28 years. A female predominance was noticed (71%). The patients were married in 75% of cases with at least one child in charge of 77% of cases. The main professional categories were nurses (29%), workers (24%), followed by anesthesia technicians and medical secretaries (8% each). The average professional seniority was 16.07 years +/- 10.34 years. Psychiatric history was found in 34.9% of the patients, 91% of whom had anxiety-depressive disorders, 4.5% bipolar disorders and 4.5% schizophrenia.

The main psychiatric reasons for the prescribed rest were characterized depressive episodes (75%), obsessive-compulsive disorder (3.2%), bipolar disorder type 2 with psychotic features (3.2%), postpartum major depressive episodes (3.2%) and post-traumatic stress disorder (3.2%). Professional conflicts with colleagues and/or superiors were reported in 21% of cases.

Psychiatric leave was prescribed by a free practice psychiatrist in 90% of cases. The average duration of leave was 50.1 days [14-180] days.

According to the opinion of the multidisciplinary commission, the healthcare professionals were considered fit to resume their professional activities in 59% of the cases, including 9 patients with restrictions (5 cases of eviction from night work, and 1 case of eviction from contact with the public, 1 case of professional reclassification and 1 case of early retirement). The leave was considered justified in 36% of cases for temporary unfitness for work.

**Conclusions:**

The medical examination of fitness for work for health care workers on psychiatric leave remains a delicate decision which can run into numerous difficulties requiring a collegial opinion from the psychiatrist and the occupational physician.

**Disclosure of Interest:**

None Declared

